# JAM-A is a novel surface marker for NG2-Glia in the adult mouse brain

**DOI:** 10.1186/1471-2202-11-27

**Published:** 2010-02-26

**Authors:** Sandra Stelzer, Klaus Ebnet, Jens C Schwamborn

**Affiliations:** 1 Westfälische Wilhelms-Universität Münster, ZMBE, Institute for Cell Biology, Stem Cell Biology and Regeneration Group, Von-Esmarch-Str. 56, 48149 Münster, Germany; 2 Westfälische Wilhelms-Universität Münster, ZMBE, Institute of Medical Biochemistry, Cell Adhesion and Cell Polarity Group, Von-Esmarch-Str. 56, 48149 Münster, Germany

## Abstract

**Background:**

Junctional adhesion molecule-A (JAM-A) is an adhesive protein expressed in various cell types. JAM-A localizes to the tight junctions between contacting endothelial and epithelial cells, where it contributes to cell-cell adhesion and to the control of paracellular permeability.

**Results:**

So far, the expression pattern of JAM-A has not been described in detail for the different cell types of the adult brain. Here we show that a subset of proliferating cells in the adult mouse brain express JAM-A. We further clarify that these cells belong to the lineage of NG2-glia cells. Although these mitotic NG2-glia cells express JAM-A, the protein never shows a polarized subcellular distribution. Also non-mitotic NG2-glia cells express JAM-A in a non-polarized pattern on their surface.

**Conclusions:**

Our data show that JAM-A is a novel surface marker for NG2-glia cells of the adult brain.

## Background

Junctional adhesion molecule-A (JAM-A, also called F11R or JAM-1) belongs to the family of junctional adhesion molecules, immunoglobulin-superfamily (Ig-SF) proteins characterized by a V-type and a C2-type Ig-like domain [1]. JAM-A is expressed mainly by epithelial, endothelial cells and certain leukocyte subsets. JAM-A undergoes homophilic binding to promote homotypic interactions between adjacent cells. In addition, it undergoes heterophilic interactions with the leukocyte integrin αLβ2 which probably serves to regulate leukocyte interactions with endothelial cells [2]. The homophilic binding is rather weak as it does not support cell adhesion of transfected cells to immobilized JAM-A Fc fusion proteins [3]. Through its cytoplasmic tail JAM-A interacts with various PDZ domain-containing scaffolding proteins, and its homophilic binding activity is proposed to regulate the specific subcellular localization of these proteins [1]. Interestingly, JAM-A directly interacts with the cell polarity protein PAR-3 [4,5], a scaffolding protein that is highly conserved through evolution and that regulates various aspects of cell polarity in different cell types including epithelial cells, neurons, neuroblasts and the *C. elegans *zygote [6]. By regulating the specific subcellular localization of PAR-3 JAM-A has been proposed to regulate the formation of tight junctions and apico-basal polarity in vertebrate epithelial cells [7]. Recently, it has been shown that JAM-A is a marker for long-term repopulating hematopoietic stem cells in adult mice [8].

The broad distribution of JAM-A and its function as a marker for adult hematopoietic stem cells prompted us to investigate JAM-A expression in the adult brain.

Neural stem cells have the characteristics of glia cells [9,10]. In the adult mammalian brain these stem cells represent a certain subtype of astrocytes [11]. However, beside astrocytes and oligodendrocytes the adult mammalian brain contains a third type of macroglia, the so called NG2-glia cells. These cells exist abundantly in the grey and white matter of the adult central nervous system (CNS) and are almost as numerous as astrocytes [12]. At least a subset of the NG2-glia cells of the adult CNS can proliferate and can function as progenitor cells for oligodendrocytes [12-15].

Here we show that JAM-A is indeed expressed in a certain population of mitotic cells in the brain. Through stainings with cell type-specific markers we identify NG-2-glia cells, and not neural stem cells or neuronal precursor cells, as the JAM-A-positive cell population. Thus, we provide evidence that JAM-A is a novel surface marker for NG2-glia cells in the brain.

## Results

### A subset of proliferating SVZ cells express JAM-A

In a first set of experiments we wanted to find out whether JAM-A is expressed in proliferating stem or progenitor cells of the adult mouse brain and whether it shows an asymmetric distribution during mitosis. The most proliferative zone of the adult mouse brain is the subventricular zone (SVZ), a region where neural stem and progenitor cells are present and where new neurons for the olfactory bulb are produced.

**Figure 1:**
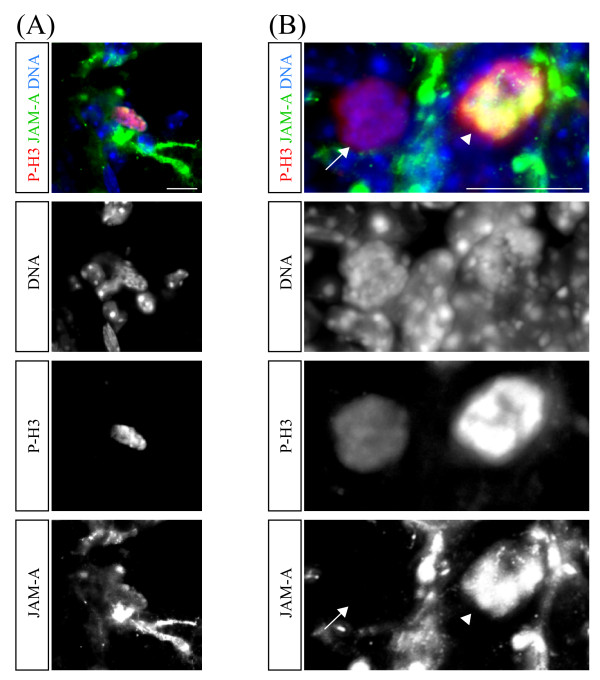
**JAM-A is expressed in a subset of proliferating cells**. Confocal images of immunostainings of vibratome sections from the subventricular zone of adult mouse brains labeled with the indicated markers (left boxes) are shown. (A) and (B) show proliferating P-H3 positive cells in two different magnifications. In (B) a maximum intensity projection of several optical layers is shown, the arrow points to a P-H3 positive and JAM-A negative cell, while the arrowhead indicates a P-H3 and JAM-A double positive cell. Scale bars: 10 μm

We identified mitotic cells in the SVZ by staining with an antibody against phosphorylated Histon H3 (P-H3). To detect JAM-A we used an anti-JAM-A antibody that is specific for just JAM-A and is not detecting other JAM-proteins like JAM-B or JAM-C [7]. Most P-H3 positive cells in the SVZ were negative for JAM-A. Interestingly, about 5% of the P-H3 positive cells were also positive for JAM-A (Figure 1). Analysis at higher magnification indicated that JAM-A is evenly distributed on the cell with no obvious asymmetric subcellular distribution (Figure 1B).

Therefore we conclude that JAM-A is expressed in a subset of proliferating cells of the adult SVZ but is not asymmetrically inherited by one of the two daughter cells after mitosis.

### JAM-A is not expressed in neural stem or progenitor cells

Next, we wanted to find out whether JAM-A expression is specific for a certain proliferating stem or progenitor cell type of the adult SVZ. If JAM-A is expressed in progenitor cells of the neuronal linage it either has to be expressed in neural stem cells (Typ-B cells), transient amplifying cells (Typ-C cells) or neuroblasts (Typ-A cells) [11]. We used antibodies against GFAP to identify neural stem cells. Because GFAP is also expressed in astrocytes, we used cellular morphology in addition to GFAP-positive staining to discriminate between neural stem cells and astrocytes. Astrocytes usually show a stellate morphology while adult neural stem cells are more spindle shaped. Therefore, only GFAP-positive cells with spindle-like morphology are classified as adult neural stem cells. Antibodies against Mash1 were used to identify transient amplifying cells, antibodies against TuJ1 and PSA-NCAM (not shown) to identify neuroblasts.

**Figure 2:**
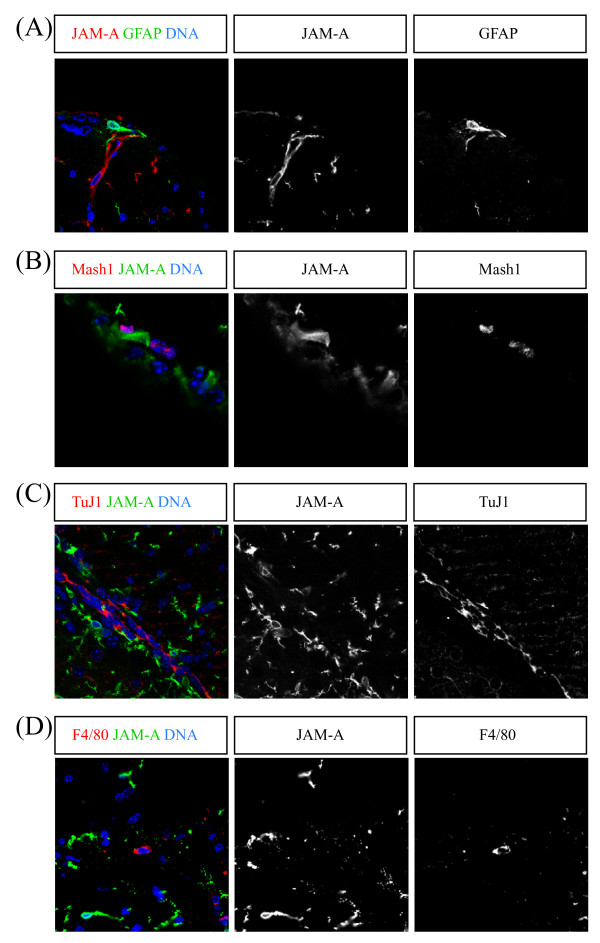
**JAM-A is not expressed in stem and progenitor cells**. Confocal images of immunostainings of vibratome sections from the subventricular zone ((A), (B) and (D)) and the rostral migratory stream (C) of adult mouse brains labeled with the indicated markers (upper boxes) are shown. To visualize GFAP expressing cells GFAP-GFP transgenic knock-in mice were used. Scale bars: 10 μm

We never observed any significant co-staining of JAM-A with GFAP, Mash1 or TuJ1 (Figure 2). These results clearly indicated that JAM-A is not expressed within stem cells or progenitor cells of the neuronal lineage in the adult brain.

Another cell type of the adult brain that is able to proliferate are microglia. Therefore we co-stained JAM-A with the microglia marker F4/80 (Figure 2D). Because no co-staining between JAM-A and F4/80 was detectable, we conclude that JAM-A is also not expressed on microglia.

### JAM-A positive cells are abundant in all areas of the adult brain

**Figure 3:**
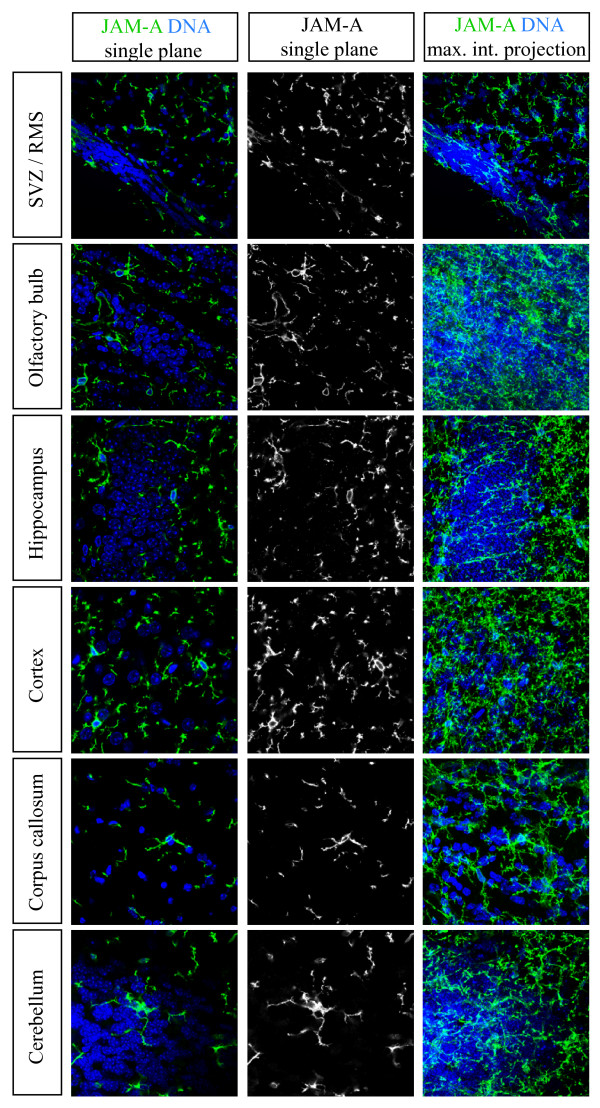
**Cells with a multiprocessed stellate morphology in different areas of the brain express JAM-A**. Confocal images of immunostainings of vibratome sections from different regions of the adult brain (left boxes) labeled with the indicated markers (upper boxes) are shown. The right column shows a maximum intensity projection of several optical layers. All JAM-A positive cells from the various indicated brain areas show the same multiprocessed stellate morphology. Scale bars: 10 μm

So far we showed that JAM-A is expressed in some proliferating cells but is not expressed in neural stem or progenitor cells. To find out how abundant JAM-A expressing cells in the adult brain are we decided to investigate the JAM-A expression in various regions of the brain. Beside a characteristic JAM-A staining for endothelial cells of blood vessels (not shown) we found that JAM-A positive cells are abundant in all grey and white matter areas of the adult brain (Figure 3). JAM-A expressing cells can be found in the subventricular zone and rostral migratory stream (RMS), the olfactory bulb, hippocampus, cortex, cerebellum and corpus callosum. Beside this abundant expression pattern it became obvious that all JAM-A positive cells in all these different regions show the same multiprocessed stellate morphology. Therefore it was likely that all JAM-A positive cells represent the same cell type.

### JAM-A is not expressed in Neurons, Oligodendrocytes or Astroglia

**Figure 4:**
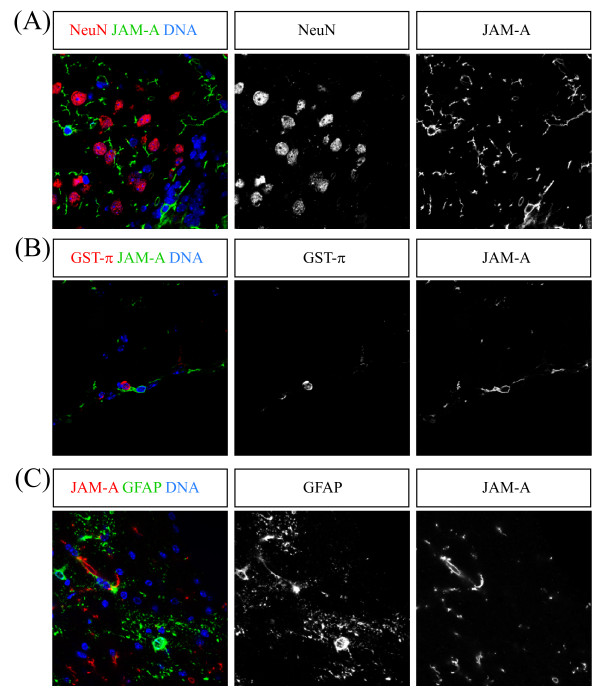
**JAM-A is not expressed in Neurons, Oligodendrocytes or Astroglia**. Confocal images of immunostainings of vibratome sections from the rostral migratory stream and adjacent brain parenchyma (A), the subventricular zone (B) and the corpus callosum (C) labeled with the indicated markers (upper boxes) are shown. JAM-A staining is not overlapping with staining for Neurons (A, NeuN), Oligodendrocytes (B, GST-π) or Astrocytes (C, GFAP). To visualize GFAP expressing cells GFAP-GFP transgenic knock-in mice were used. Scale bars: 10 μm.

To find out which of the major cell lineages of the adult brain expresses JAM-A we used markers for Neurons (NeuN), oligodendrocytes (GST-π) and astrocytes (GFAP). However, we never found any significant co-staining between JAM-A and NeuN, GST-π or GFAP (Figure 4). Therefore we conclude that JAM-A positive cells are neither neurons, nor astrocytes nor oligodendrocytes.

### JAM-A is a specific surface marker for NG2-Glia cells

**Figure 5:**
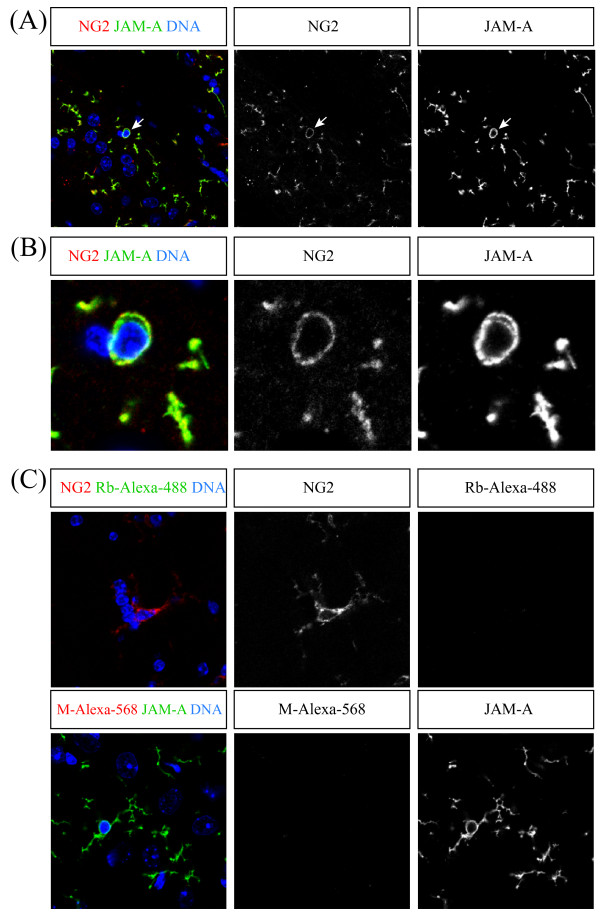
**NG2-glia cells express JAM-A**. Confocal images of immunostainings of vibratome sections from the corpus callosum labeled with the indicated markers (upper boxes) are shown. In (B) the cell body labeled with an arrow in (A) is shown at higher magnification. In (C) negative controls, where only one primary antibody but the two secondary antibodies anti-mouse-Alexa-568 nm (M-Alexa-568) and anti-rabbit-Alexa-488 nm (Rb-Alexa-488) have been used, are shown. Scale bars: 10 μm

**Figure 6:**
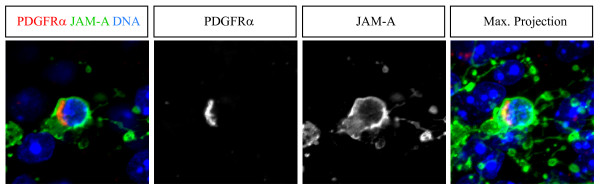
**PDGFRα positive NG2-glia cells express JAM-A**. Confocal images of immunostainings of vibratome sections from the corpus callosum labeled with the indicated markers (upper boxes) are shown. The last panel shows a maximum intensity projection of several optical sections. Scale bars: 10 μm

Beside astrocytes and oligodendrocytes the adult mammalian brain contains a third type of macroglia, the so called NG2-glia cells [12,13]. These cells are called NG2-glia cells because they express the glycoprotein NG2. Because these cells also show a multiprocessed stellate morphology as observed for JAM-A expressing cells (Figure 3) we co-stained for NG2 and JAM-A. In all brain regions investigated more than 90% (95,5% +/- 4%) of the JAM-A positive cells were also positive for NG2 (Figure 5). Additionally, nearly all NG2 positive cells were also positive for JAM-A (96,4% +/- 6%). However, NG2 is also expressed on pericytes. Therefore we used the PDGF-alpha receptor as additional NG2-glia marker [12]. Conclusively, we found a strong co-expression of JAM-A and the PDGF-alpha receptor (Figure 6). Previously it has been described that the GFAP-GFP mice used in this study show a weak expression of GFP in NG2-glia [16]. However, with our staining procedure we never found a co-staining of GFAP-GFP and NG2 or GFAP-GFP and JAM-A.

From these observations, we conclude that JAM-A is a novel specific surface marker for NG2-glia of the adult mouse brain.

## Discussion

In this study we have analyzed the expression profile of the cell surface molecule JAM-A in the adult brain. We find that JAM-A is expressed on a subset of proliferating cells in the SVZ. To our surprise, these cells turned out to belong to the lineage of NG2-glia cells but not to the lineages of neural stem or progenitor cells (negative for the marker GFAP, Mash1 and TuJ1) or microglia (negative for F4/80). Thus, our findings indicate a cell type-specific expression pattern of JAM-A in the adult brain.

During asymmetric cell divisions, certain proteins are unevenly distributed among the two daughter cells [9,10,17]. This asymmetric distribution depends on their polarized subcellular distribution in the dividing cell. The Junctional adhesion molecule-A (JAM-A) is well known to show such a polarized enrichment on the apical pole of various polarized cell types [1,18-20]. This opened the possibility that JAM-A is asymmetrically distributed during cell divisions. However, we found that JAM-A is evenly distributed in proliferating cells (Figure 1), which makes it likely that JAM-A is also equally inherited by both daughter cells and therefore is not a cell fate determinant. The role of JAM-A during cell division is not known. It is interesting to note that JAM-A has recently been found to regulate cell proliferation in epithelial cells. Genetic ablation of JAM-A in mice increased the proliferation of cells in colonic mucosal epithelial cells [21]. These findings further support a role of JAM-A in the regulation of cell proliferation although the molecular mechanisms underlying this function of JAM-A still need to be revealed. JAM-A is known to be expressed by various cell types including epithelial cells, endothelial cells, hepatocytes, various leukocyte subsets, platelets, Sertoli cells, spermatozoa and hematopoietic stem cells [1,8,18-20]. It is thus not surprising that various functions have been attributed to JAM-A. One major function of JAM-A is the regulation of inflammatory responses by mediating specific interactions between leukocytes and endothelial cells [2]. Here, for the first time, we described the expression of JAM-A on cells from the adult central nervous systems.

In the adult, the discovery of functional receptors, interactions with neurons and ability to respond to different harmful stimulations have implied roles of NG2 cells in facilitating neuronal network function, which may be important in brain inflammation, neurodegeneration and neuroregeneration [22]. Therefore, it seems possible that JAM-A is involved in homotypic NG2-glia cell interactions or heterotypic interactions between NG2-glia cells and other cell types in the CNS through as yet unidentified receptors on these cells. Whether JAM-A is indeed mediating such cell-cell interactions in the central nervous system, like it has been shown for platelets and endothelial cells [23,24], is currently unknown. In addition, JAM-A could also influence the adhesion of the NG2-glia cells to the extracellular matrix and thereby regulate the migration of the NG2-glia cells within the CNS. A role of JAM-A in cell migration by regulating the levels and/or activity of integrins has been described in epithelial cells, endothelial cells and neutrophil cells [25-28]. A second major function of JAM-A is the regulation of tight junction formation and of apico-basal polarity development in vertebrate epithelial cells [1]. This function of JAM-A resides in its ability to interact with the cell polarity protein PAR-3 and to govern the specific subcellular localization of PAR3 and the PAR3-associated aPKC - PAR6 complex during junction formation [7,29]. Whether the PAR3-PAR6-aPKC complex is regulated by JAM-A in NG-2 glia cells remains to be shown.

## Conclusions

In summary, here we describe for the first time the expression of JAM-A on cells from the adult nervous system. JAM-A is exclusively expressed by a specific subtype of macroglia, the so-called NG2-glia cells.

## Methods

### Materials

The following primary antibodies were used: anti-JAM-A (rabbit) [7], anti-P-H3 (mouse, New England Biolabs), anti-GFAP (mouse, Millipore), anti-Mash1 (mouse, BD Bioscience), anti-TuJ1 (mouse, Covance), anti- GST-π (mouse, BD Bioscience), anti-NG2 (mouse, Millipore), anti-PDGFRα (rat, Millipore) and F4/80 (rat, Abcam). As secondary antibodies Alexa-fluorophore conjugated antibodies (goat, Invitrogen) were used. For staining of DNA Hoechst 33258 (Invitrogen) was used.

### Mice

Mice were kept under standard conditions according to governmental rules and regulations. All stainings were repeated on sections from at least three different mice. The age of mice was between 60 and 90 days.

### Immunohistochemistry

Adult mouse brains were fixed via perfusion with 120 mM phosphate buffer, pH 7.4 (PBS) followed by perfusion with 4% paraformaldehyde in PBS. After dissection the brains were post-fixed over night with 4% paraformaldehyde in PBS. Sagittal sections (40 μm) were prepared with a Vibratome (Leica). Sections were block with blocking buffer (100 mM Tris buffer, 0.5% Triton X-100, 0.1% sodium azide, 0.1% sodium citrate and 5% normal goat serum). Blocking was followed by incubation with primary and secondary antibodies, both diluted in the blocking solution. Images were collected by confocal microscopy using CEN software (Zeiss); image analysis was performed with the CEN software, Adobe Photoshop and the IMAGE J software.

All experiments involving mice have been conducted according to German Animal Welfare Act and have been approved by the responsible authorities (Landesamt für Natur, Umwelt und Verbraucherschutz Nordrhein-Westfalen).

## Competing interests

The authors declare that they have no competing interests.

## Authors' contributions

S.S. performed immunostainings, did all the tissue preparations and was involved in the microscopic analysis. K.E. was involved in the experiment planning, manuscript writing and picture analysis. J.C.S. did the microscopic analysis, supervised and planned all the experiments and wrote the manuscript.

All authors discussed and commented on the findings in the manuscript and improved writing.
